# A37 EFFICACY AND SAFETY OF RISANKIZUMAB AS MAINTENANCE THERAPY IN PATIENTS WITH CROHN’S DISEASE: 52 WEEK RESULTS FROM THE PHASE 3 FORTIFY STUDY

**DOI:** 10.1093/jcag/gwab049.036

**Published:** 2022-02-21

**Authors:** R Panaccione, M Ferrante, B G Feagan, W Sandborn, J Panes, L Peyrin-Biroulet, J Colombel, S Schreiber, M Dubinsky, F Baert, T Hisamatsu, E Neimark, B Huang, X Liao, A Song, S Berg, W Duan, Y Pang, V Pivorunas, K Kligys, K Wallace, G D’Haens

**Affiliations:** 1 University of Calgary, Calgary, AB, Canada; 2 Universite de Lorraine, Nancy, Lorraine, France; 3 Mount Sinai Health System, New York, NY; 4 Alimentiv Inc, London, ON, Canada; 5 University of California San Diego, La Jolla, CA; 6 Katholieke Universiteit Leuven Universitaire Ziekenhuizen Leuven Campus Gasthuisberg, Leuven, Flanders, Belgium; 7 Institut d’Investigacions Biomediques August Pi i Sunyer, Barcelona, Catalunya, Spain; 8 Universitatsklinikum Schleswig-Holstein, Kiel, Schleswig-Holstein, Germany; 10 AZ Delta vzw, Roeselare, West-Vlaanderen, Belgium; 11 Kyorin Daigaku Igakubu Daigakuin Igaku Kenkyuka, Mitaka, Tokyo, Japan; 12 AbbVie Inc, North Chicago, IL; 13 Universiteit van Amsterdam, Amsterdam, Noord-Holland, Netherlands

## Abstract

**Background:**

Risankizumab (RZB), an anti-IL-23 p19 inhibitor, was well-tolerated and superior to placebo (PBO) in inducing clinical remission and endoscopic response in patients (pts) with moderate-to-severe Crohn’s disease (CD) in two phase 3 studies at 12 weeks.

**Aims:**

FORTIFY (NCT03105102), was a 52-week (wk) phase 3 double-blind, re-randomized responder withdrawal study that evaluated the efficacy and safety of continuing RZB as subcutaneous (SC) maintenance therapy versus withdrawal to placebo in pts achieving induction response to RZB

**Methods:**

Week 12 IV RZB responders were re-randomized 1:1:1 to: RZB SC 360mg (N=141), RZB 180mg (N=157), or PBO (withdrawal from IV RZB; N=164) every 8wks for 52wks. Co-primary endpoints were clinical remission (per CD Activity Index [CDAI] (US); or stool frequency/abdominal pain score [SF/APS] (OUS) and endoscopic response at wk52. Other clinical and endoscopic endpoints, inflammatory biomarkers, RZB serum levels, and safety were assessed over time.

**Results:**

Rates of clinical remission (CDAI, SF/APS) and clinical response were similar for RZB and PBO groups through wk24, with rates lower for PBO thereafter. At wk52, clinical remission (CDAI, SF/APS) and endoscopic response rates were significantly higher with RZB 360mg than PBO ( *P*<0.01); RZB 180mg was superior to PBO for clinical remission per CDAI and endoscopic response ( *P*<0.01). Endoscopic remission and deep remission rates increased over time with 360mg, remained steady with 180mg, and decreased with PBO. Mean fecal calprotectin (FCP) and C-reactive protein (CRP) levels decreased with SC RZB, but increased with PBO, over 52wks. Exposure-adjusted event rates (per 100 pts-years) of serious adverse event (AE) were generally similar among groups (360mg, 21.0 E/100PY and 180mg, 19.5 E/100PY vs PBO, 19.3 E/100PY), as were AEs leading to drug discontinuation (4.8 E/100PY and 2.4 E/100PY vs 3.7 E/100PY), and serious infections (6.0 E/100PY and 3.0 E/100PY vs 5.0 E/100PY).

**Conclusions:**

In pts with moderate-to-severe CD, a robust pharmacodynamic effect on the IL-23 pathway after 12wks RZB IV induction was maintained with RZB SC maintenance therapy. The durability of RZB was demonstrated with high rates of efficacy over the 52-wk study. RZB was superior to PBO for achieving clinical remission and endoscopic response at wk52. Results for the more stringent endpoints (endoscopic remission\deep remission) and persistent improvements in inflammatory biomarkers are consistent with a dose response relationship. Continued RZB SC maintenance treatment was generally safe and well-tolerated.

**Funding Agencies:**

AbbVie

